# Comparative Effects of Aminophylline, Caffeine, and Doxapram in Hypoxic Neonatal Dogs Born by Cesarean Section

**DOI:** 10.3390/ani15233485

**Published:** 2025-12-03

**Authors:** Júlia Cosenza Mendonça, Keylla Helena Nobre Pacífico Pereira, Gleice Mendes Xavier, Kárita da Mata Fuchs, Thaís Gomes Faustino, Viviane Maria Codognoto, Miriam Harumi Tsunemi, Regina Kiomi Takahira, Maricy Apparício, Maria Lucia Gomes Lourenço

**Affiliations:** 1 Department of Veterinary Clinics, School of Veterinary Medicine and Animal Science, São Paulo State University (Unesp), Botucatu 18618-970, Brazil; julia.cosenza@unesp.br (J.C.M.); gleice.xavier@unesp.br (G.M.X.); karita.fuchs@unesp.br (K.d.M.F.); thais.faustino@unesp.br (T.G.F.); viviane.codognoto@unesp.br (V.M.C.); regina.takahira@unesp.br (R.K.T.); maricy.apparicio@unesp.br (M.A.); 2 Department of Veterinary Medicine, Federal University of Alagoas (UFAL), Viçosa 57700-000, Brazil; keylla.pereira@vicosa.ufal.br; 3 Department of Biostatistics, Institute of Biosciences of Botucatu, São Paulo State University (Unesp), Botucatu 18618-689, Brazil; m.tsunemi@unesp.br

**Keywords:** canine neonatology, neonatal resuscitation, hypoxia, Apgar score, hypoxia-induced depression, perinatal viability, respiratory stimulants, methylxanthines

## Abstract

Background: Perinatal hypoxia is a leading cause of early mortality in canine neonates and accounts for over 90% of early deaths in canine neonates. Respiratory depression may result from dystocia, prolonged labor, or anesthetic protocols used during cesarean delivery, requiring rapid pharmacological support. This study evaluated the effects of aminophylline, caffeine, and doxapram on clinical and biochemical viability parameters in hypoxic neonatal dogs. Methods: Forty-five puppies born via elective or emergency cesarean section were included, of which 35 exhibiting cardiorespiratory depression (heart rate ≤ 180 bpm, respiratory rate < 15 movements per minute, Apgar score < 7, and reflex score < 3) were randomly allocated into aminophylline group (AG; n = 12), caffeine group (CafG; n = 11), or doxapram group (DG; n = 12). Ten clinically healthy neonates constituted the physiological control group (CG). Apgar score, neonatal reflexes, lactate, blood glucose, oxygen saturation, blood gas parameters, and cardiac troponin I (cTnI) were evaluated immediately after birth (M1) and 10 min following treatment (M10). Results: No significant differences were observed in laboratory parameters among groups (*p* > 0.05). Although all evaluated drugs improved clinical status by increasing heart rate, respiratory rate, and reflex scores, caffeine produced the greatest enhancement in vitality. The Δ Apgar score showed a significant difference between CafG and DG (*p* = 0.0095), while no statistical differences were detected between AG and CafG (*p* = 0.08) or between AG and GD (*p* = 0.60). Conclusions: Aminophylline, caffeine, and doxapram supported postnatal resuscitation in hypoxic neonatal puppies; however, caffeine demonstrated superior improvement in Apgar recovery, suggesting its potential as a preferred pharmacological adjunct in neonatal canine resuscitation protocols.

## Simple Summary

Hypoxia, a reduction in tissue oxygen supply, is a critical condition that may occur during birth, especially in cases of dystocia or poorly managed cesarean sections. This condition can severely affect the survival and early development of newborn puppies. This study aimed to compare the effectiveness of three respiratory stimulants—aminophylline, caffeine, and doxapram—in hypoxemic neonatal dogs delivered by cesarean section. Forty-five hypoxic puppies were randomly assigned to one of the three treatment groups, while a separate group of healthy neonates served as a control. However, one limitation was the absence of a placebo control group. Clinical and laboratory parameters were evaluated at birth and 10 min after drug administration. Although no significant changes were observed in laboratory variables within or between treatment groups, caffeine showed a greater improvement in Apgar scores compared with doxapram. This suggests that caffeine may provide superior immediate clinical recovery in hypoxemic newborn puppies. Based on these findings, caffeine may be considered a preferable first-line pharmacological option for supporting respiratory stimulation in neonatal puppies following cesarean delivery, potentially improving early vitality and aiding clinical decision-making in neonatal resuscitation.

## 1. Introduction

Neonatal mortality remains a major concern in canine neonatology, with rates significantly higher than those reported in human medicine. While global human neonatal mortality averages approximately 1.9%, and 1.51% in Brazil, mortality in canine neonates may range from 6.9% to 35%, particularly during birth, immediately postpartum, or within the first week of life [1,2,3,4]. This high mortality rate is frequently associated with unrecognized or underestimated perinatal risk factors.

The fetal-to-neonatal transition is a critical physiological event in which the neonate must rapidly assume vital functions previously maintained by the placenta, including independent pulmonary respiration [5]. Failure in this transition, often due to perinatal asphyxia, may result in hypoxemia and subsequent hypoxia, leading to respiratory distress syndrome, metabolic and respiratory acidosis, and tissue damage in organs with high oxygen demand [6,7,8].

Among hypoxia-sensitive organs, the myocardium is particularly vulnerable to oxygen deprivation, and cardiac troponin I (cTnI) has been investigated as a biomarker of myocardial injury in neonates. Previous findings from our research group demonstrated the applicability of cTnI in neonatal dogs with perinatal asphyxia, supporting its use as a marker of myocardial stress in hypoxic conditions [9,10].

Cesarean delivery, dystocia, and prolonged labor are commonly associated with neonatal respiratory depression and increased risk of hypoxic injury [11,12]. According to the 2025 RECOVER Guidelines for newborn resuscitation in dogs and cats, in such cases, rapid and effective neonatal resuscitation is essential to reestablish oxygen delivery, enhance cardiopulmonary function, and improve viability outcomes. Pharmacological support has been proposed as an adjunct to mechanical stimulation and oxygen supplementation in neonatal resuscitation protocols [13,14].

Respiratory stimulants such as aminophylline, caffeine, and doxapram have been used in both human and veterinary medicine to enhance respiratory drive through central nervous system and chemoreceptor activation [15,16,17,18]. However, comparative studies evaluating the efficacy of these agents in hypoxic neonatal dogs are limited, and evidence-based recommendations for their clinical application remain scarce.

Therefore, this study aimed to evaluate the effects of aminophylline, caffeine, and doxapram on clinical and biochemical viability parameters in hypoxic neonatal dogs born via elective or emergency cesarean section.

## 2. Materials and Methods

### 2.1. Ethical Approval

The study protocol was reviewed and approved by the Animal Use Ethics Commi tee (CEUA), School of Veterinary Medicine and Animal Science—UNESP, Botucatu, Brazil (protocol number 000.103) and after the tutor’s authorization and the signing of the free and informed consent form. All procedures complied with Brazilian regulations for animal experimentation. To minimize bias in the research and increase the validity of the results, this study was blinded; the individual responsible for administering the treatments was unaware of the drug identity and group allocation.

### 2.2. Animals and Experimental Procedure

The study was conducted at the Small Animal Reproduction Service, School of Veterinary Medicine and Animal Science (UNESP), Botucatu, Brazil.

The study included 14 adult bitches of different breeds and ages that required cesarean delivery, as well as their neonates. The inclusion criteria for female dogs in the study included healthy bitches, up to four years of age, and for elective cesarean section, data on the date of mating or artificial insemination. The exclusion criterion was bitches with infections.

Seven females underwent elective cesarean section as they were brachycephalic breeds. The following criteria were used to determine the timing of the surgery: gestation period (58 to 63 days after the first mating or artificial insemination); ultrasound findings (reduced amniotic fluid, presence of peristalsis and reduced fetal heart rate, measurement of the parietal diameter to estimate gestational age) [19]; reduced rectal temperature of the bitch; assessment of behavioral and clinical changes consistent with impending parturition, such as anorexia, nest preparation, elimination of the mucous plug and restlessness; and determination of serum progesterone concentration by quantitative chemiluminescence assays, performing the cesarean section when the levels of this hormone reached values ≤ 2 ng/mL [20].

Seven females underwent emergency cesarean section due to fetal distress observed by ultrasound or when they did not respond to pharmacological therapy or obstetric maneuvers performed to correct maternal or fetal dystocia (such as secondary uterine atony and fetal incarceration in the vaginal canal). Two females received 10% calcium gluconate at a dose of 1 mg/kg and 10% glucose at a volume of 0.5 mL/kg [21]. In one female, an attempt at retropulsion and fetal traction was performed. Cesarean sections were performed in a standardized manner by specialist animal reproduction surgeons at the veterinary hospital.

Anesthetic protocol for cesarean sections [22] was performed with induction with intravenous propofol at a dose sufficient to cause loss of the laryngotracheal reflex. Then, epidural anesthesia was performed with 2% lidocaine, 4 mg/kg, single dose. Anesthetic maintenance was performed with 1% of isofluorane diluted in 100% oxygen, which was administered through a circular valve anesthetic circuit. After removing the puppies from the uterus, fentanyl (5 µg/kg, single dose) was administered intravenously to the bitches.

During the cesarean delivery, fetuses were separated from the placenta while maintaining the umbilical cord intact, which was only clamped and cut after neonatal stabilization. Neonatal assistance was provided immediately after birth.

Immediately after birth, the neonates were kept on a heated surface, where stimulation for neonatal breathing was performed, including clearing the airways with the aid of a pediatric bulb syringe and chest rubbing with the help of a cloth.

The inclusion criteria for hypoxic neonates was a clinical diagnosis of cardiorespiratory depression (heart rate ≤ 180 bpm, respiratory rate < 15 mpm, Apgar score < 7, and reflex score < 3), associated with blood gas parameters at birth [1,8,9].

The inclusion criterion for non-hypoxic neonates was neonates that did not require resuscitative or respiratory support, presenting eupnea, heart rate between 200 and 260 bpm, respiratory rate between 15 and 40 mpm, pink mucous membranes, and Apgar score ≥ 7 within five minutes after birth; associated with blood gas parameters at birth [1,8,9]. Therefore, this group served as a physiological reference pattern rather than pharmacological control.

The exclusion criteria were neonates who showed signs of systemic infection (meconium diarrhea, abdominal erythema, omphalitis, cyanosis of the extremities), congenital malformations, and signs of prematurity (absence of hair on limbs and snout) [23,24].

### 2.3. Grouping and Treatment Protocol

At birth (within the first five minutes), the neonates were assessed for Apgar score, reflexes, pulse oximetry, and a blood sample was collected for blood gas analysis, lactate, blood glucose, and troponin I measurements. Following this preliminary assessment, 45 neonates were included in the study; of these, 35 neonates exhibiting hypoxia were randomly allocated into aminophylline (AG) n = 12, caffeine (CafG) n = 11, or doxapram (DG) n = 12. Ten non-hypoxic neonates were allocated into Control Group (CG).

**Figure 1:**
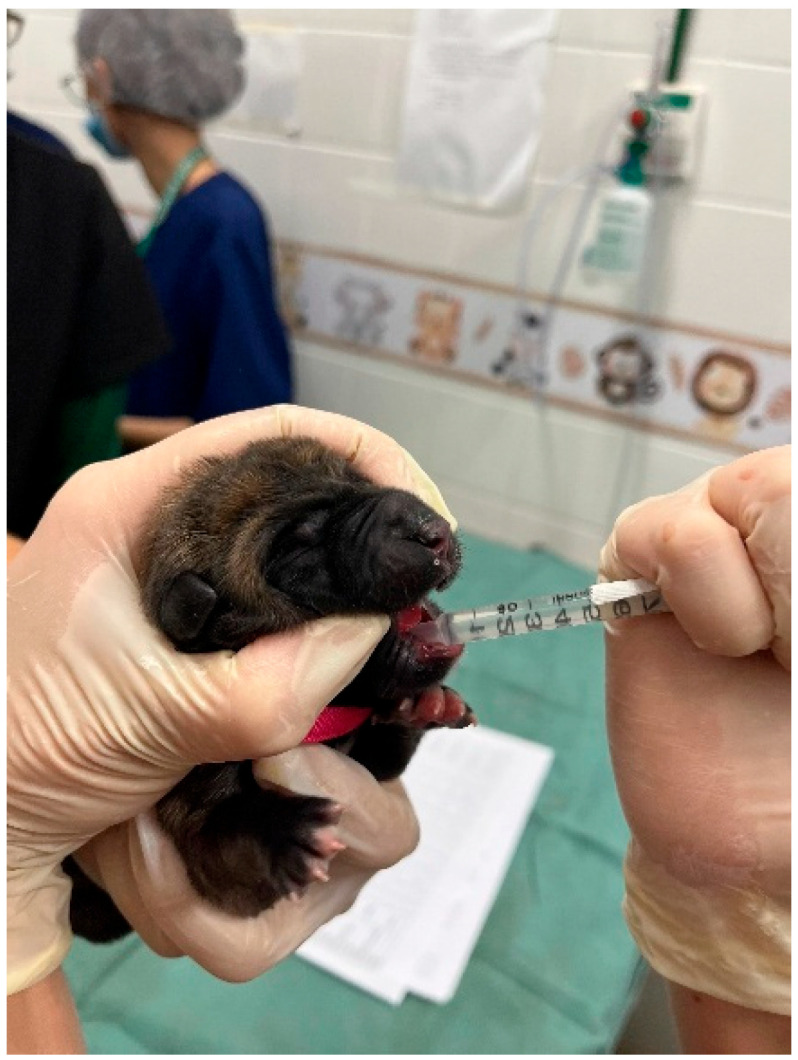
Sublingual administration of pharmacological treatment in hypoxic neonatal dogs during the resuscitation protocol.

In the Aminophylline Group (AG), neonates received sublingual administration (Figure 1) of aminophylline (24 mg/mL; Teuto^®^, Anápolis, GO, Brazil) at a dose of 0.2 mL/100 g of body weight. The Caffeine Group (CafG) received sublingual caffeine (25%; Biofarm^®^, Jaboticabal, SP, Brazil) at a dose of 0.1 mL/100 g, and the Doxapram Group (DG) received sublingual doxapram (20 mg/mL; Holliday-Scott^®^, Béccar, Argentina) at a dose of 0.1 mL/100 g of body weight.

Clinical and laboratory parameters were evaluated two times: M1, corresponding to five minutes after birth (prior to drug administration), and M10, corresponding to ten minutes after the drug administration.

### 2.4. Clinical and Biochemical Parameters Evaluated

Only one individual assessed all the neonates, avoiding variations in assessment methods between different people. Because the study was blinded, the parameter assessor did not know which medication was administered to each neonate evaluated.

The evaluation of neonatal viability was similar in the three groups. We evaluated the modified Apgar score [1,25]. The evaluation of heart rate was performed using a vascular Doppler ultrasound. The respiratory frequency and breathing patterns of the neonate were noted, and reflex irritability was checked by a painful stimulus. Muscle tone was determined with the neonate in a supine position by observing active movements and responses to passive movements of the limbs. The appearance of the mucous membranes was assessed by visualization of the oral mucosa. Each parameter was scored on a scale from 0 to 2, and the total score was the sum of these (Table 1).

Neonatal reflexes (suckle, rooting, and righting reflexes) were also assessed at birth and 10 min later (Table 2). The suckle reflex was elicited by inserting the clean tip of the smallest digit of the examiner into the mouth of the neonate and assessing the suckling force; the righting reflex of the neonate was assessed by placing it on its back on a surface and verifying whether it returned to sternal recumbency. The rooting reflex was assessed by approaching the nose of the neonate with a hand shaped into a circle with the thumb and forefinger and checking whether the neonate inserted its nose into the circle. The reflexes were scored on a scale from 0 to 2, and the total score was their sum. Joint analysis of all reflexes was used because the presence of one reflex alone does not ensure neonatal survival. The interpretation of the score was as follows: 0–2, weak viability; 3 and 4, moderate viability; and 5 and 6, normal viability [1,25]. Weight in grams was assessed with a small digital scale.

After determination of the Apgar score and neonatal reflexes, a blood sample (0.3 mL) was drawn by jugular puncture (0.1 mL for use in the CG4+ cartridge; 0.1 mL for the cTnI cartridge; 0.1 mL for the portable blood glucose meter) witha1-mL syringe and a 26-G needle. The blood volume of newborns is approximately 5 to 8 mL/100 g of body weight. The blood sample collected must be less than 1 mL/100 g of weight in 24 h [20]. All laboratory assessments were performed at the site of the birth, and the results were used to determine whether the newborns required emergency treatment.

The evaluations were performed with an i-STAT Portable Clinical Analyzer (Abbott Laboratories, Abbott Park, Illinois, IL, USA) with an CG4+ cartridge for blood gas analysis (ABBOTT) on venous blood samples obtained via jugular puncture. The following parameters were assessed: blood pH, partial pressure of oxygen and carbon dioxide (pO_2_ and pCO_2_, mmHg), base excess (BE, mmol/L), sodium bicarbonate (HCO_3_, mmol/L), oxygen saturation (sO_2_%), and lactate (mmol/L). Peripheral oxygen saturation (SpO_2_%) was assessed using an oximetry monitor (R40 VET, Rzvet^®^, Curitiba, PR, Brazil) placed in the femoral artery region. A determination of blood glucose levels was performed with a portable blood glucose meter (On Call Plus^®^, San Diego, CA, USA). The parameters were interpreted according to the reference values reported for neonatal puppies at birth (Table 3) [1,8,9].

**Figure 2:**
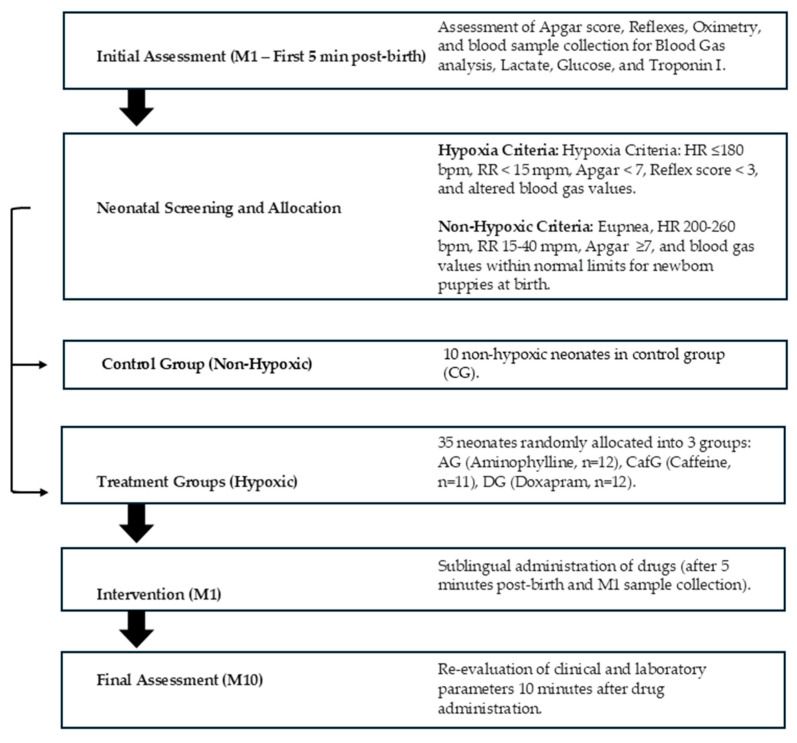
Flowchart of the screening, group allocation, and puppy evaluation steps.

cTnI analyses were performed using a disposable cartridge (cTnI-specific kit) developed for use in a portable clinical analyzer (i-Stat 1^®^ Portable Clinical Analyzer, Abbott Laboratories, Abbott Park, Illinois, IL, USA). Levels were interpreted according to reference values reported for adult dogs (<0.006–0.05 ng/mL) [10] and based on previously published findings from our research group evaluating cTnI as a biomarker of severe hypoxia and myocardial injury in neonatal puppies subjected to perinatal asphyxia (Table 3) [9]. A flowchart summarizes the study methodology (Figure 2).

### 2.5. Statistical Analysis

Data were organized using Microsoft Excel, and statistical analyses were performed using R 2025. The normality of data distribution was assessed using the Shapiro–Wilk test, while homogeneity of variances was verified using Levene’s test. For variables that met the assumptions of normality and homoscedasticity, comparisons among treatment groups (aminophylline, caffeine, and doxapram) were conducted using one-way ANOVA, followed by Tukey’s post hoc test when applicable. For non-normally distributed data, the Kruskal–Wallis test was applied, followed by Dunn’s multiple comparison test using appropriate R statistical functions.

Inferential analyses were conducted using available case data. Due to insufficient sample volume or invalid readings in some cases, the sample size (n) varied across outcomes and treatment groups. The difference between time points (Δ = M10 − M1) was calculated only for neonates with paired measurements; therefore, puppies without M10 data (e.g., early death) were excluded from Δ-based analyses. No data imputation procedures were applied. The control group, consisting of physiologically normal neonates, was included only as a descriptive reference and was not subjected to inferential comparisons.

Data are presented in tables as mean ± standard deviation (SD) for normally distributed variables or as median and interquartile range (IQR) for non-parametric variables. The exact sample size used in each comparison is reported in the corresponding table headers or footnotes. Statistical significance was set at *p* < 0.05. All analyses were performed using two-tailed tests, and results are reported with 95% confidence intervals where applicable.

## 3. Results

A total of 69 neonatal puppies born from 14 bitches delivered by cesarean section were recorded, of which 45 met the inclusion criteria and were enrolled in the study. The mean litter size, calculated including all live-born pups per bitch, was 4.93 ± 3.63 puppies. The maternal population had a mean age of 2.64 ± 0.93 years and a mean body weight of 12.64 ± 9.18 kg.

Among the 45 included neonates, the mean birth weight was 267.28 ± 97.34 g, and mean rectal temperature at birth was 33.45 ± 1.65 °C. The sex distribution comprised 22 females (48.8%) and 23 males (51.1%). Three early neonatal deaths (7.0%) were recorded overall, including two from the aminophylline group and one from the caffeine group (Table 4). Twenty-four neonates were excluded from the study; two presented congenital malformation (cleft palate) and 22 were puppies that were born with stable parameters but were not included in the study because the control group was already complete.

The sample included 14 bitches of eight different breeds, predominantly French Bulldogs (28.6%), followed by Dachshunds (14.3%), Pinschers (14.3%), and mixed-breed dogs (14.3%). Less frequent breeds included American Bully (7.1%), Pastor Malinois (7.1%), Pug (7.1%), and Cavalier (7.1%).

A physiological reference control group was used to establish normal baseline values for neonatal dogs that did not require resuscitation or pharmacological support.

Recovery of the clinicals parameters, Apgar score, neonatal reflexes and peripheral oxygen saturation (SpO_2_%) was observed before and after treatment, are shown in Table 5.

No statistically significant differences were observed between moments M1 and M10 within each treatment group (aminophylline, caffeine, and doxapram) for any of the evaluated clinical variables. Although mean improvements were detected in parameters such as heart rate, Apgar score, and neonatal reflex responses over time, these within-group variations did not reach statistical significance (Table 6). Paired M1–M10 data were available for 12, 11, and 12 puppies in the aminophylline, caffeine, and doxapram groups, respectively. None of the neonates required additional oxygen therapy following pharmacological administration, indicating adequate spontaneous respiratory recovery after initial stimulation.

**Figure 3:**
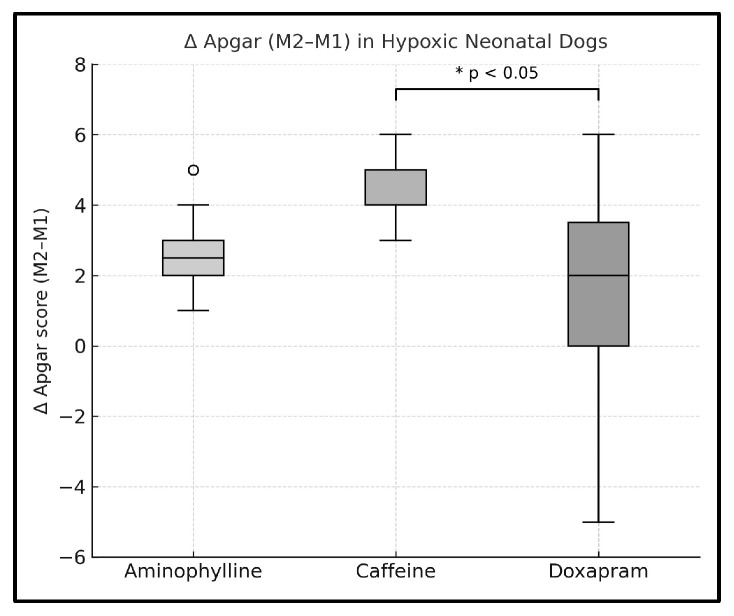
Boxplot of Δ Apgar score (M10 − M1) in hypoxic neonatal dogs treated with aminophylline, caffeine, or doxapram. Data are expressed as individual values with distribution (median and interquartile range). Caffeine-treated neonates showed significantly greater improvement compared with the doxapram group (* *p* < 0.05, Kruskal–Wallis followed by Dunn’s post hoc test).

In the intergroup comparison, a statistically significant difference was observed only for the Δ in total Apgar score. Analysis of variance (ANOVA) revealed a significant overall difference among treatment groups (*p* = 0.011), which was corroborated by the Kruskal–Wallis test (*p* = 0.009). Post hoc comparisons showed a statistically significant difference between the caffeine and doxapram groups (*p* = 0.009), whereas no significant differences were detected between aminophylline and caffeine (*p* = 0.084) or between aminophylline and doxapram (*p* = 0.608) (Table 7 and Figure 3). In the doxapram group, two neonates died before the M10 evaluation and were therefore excluded from the Δ analysis. Thus, Δ-based comparisons were conducted using available paired observations only.

Detailed laboratory descriptive statistics for all evaluated variables at both time points across groups are presented in Table 8.

Overall, none of the blood gas or biochemical parameters showed significant intragroup changes between M1 and M10 within each treatment group. These findings indicate that the pharmacological interventions did not acutely influence systemic metabolic or gas exchange parameters over the short observational period.

## 4. Discussion

This study aimed to compare the effects of aminophylline, caffeine, and doxapram on clinical recovery in hypoxic neonatal dogs. In this context, neonatal vitality remains a determining factor for early survival in canine neonates, particularly during the perinatal transition when cardiopulmonary adaptation is highly dependent on adequate respiratory effort and tissue oxygenation [1,8,9,25,26]. In the present study, the administration of respiratory stimulants in hypoxic neonates resulted in clinical improvement across all treatment groups, as indicated by increases in Apgar and reflex scores between baseline and 10 min post-intervention. Although these changes did not reach statistical significance in intragroup comparisons, the overall trend toward progressive recovery suggests partial responsiveness to drug administration in the acute postnatal period. These findings align with previous reports indicating that early pharmacological support, when combined with standard resuscitative procedures, may contribute to improving short-term clinical stability in neonates with respiratory depression [13,14,16,27,28].

According to the 2025 RECOVER Guidelines for newborn resuscitation in dogs and cats, stabilization efforts should initially focus on airway clearance, thermal support, and assisted ventilation, while pharmacological agents are recommended only as adjunctive measures when spontaneous respiratory effort remains insufficient [13]. The accompanying Evidence and Knowledge Gap Analysis further emphasizes the scarcity of robust, comparative data on the use of respiratory stimulants in veterinary neonates, particularly regarding their efficacy in cases of perinatal hypoxia [14]. In this context, the present study provides relevant comparative evidence that contributes to filling part of this knowledge gap, particularly with respect to the superior clinical responsiveness associated with caffeine when compared to aminophylline and doxapram.

When evaluated in the context of previously published data, the clinical response pattern observed in this study aligns with findings in both veterinary and human neonatology. Santos et al. [16] demonstrated that aminophylline resulted in superior Apgar recovery compared with doxapram in neonatal dogs born via cesarean section, particularly when administered sublingually. In our study, caffeine promoted improved clinical vitality, surpassing doxapram in terms of Δ Apgar response. These results reflect those of Schmidt et al. [17] who reported that caffeine provided a more sustained respiratory stimulant effect and better survival outcomes than aminophylline in preterm human infants. Thus, the enhanced performance of caffeine observed in the present study supports its classification as the most pharmacodynamically advantageous methylxanthine for neonatal respiratory stimulation. These findings are further contextualized when compared with previously published data across different veterinary and medical models. As summarized in Table 9.

Aminophylline, another methylxanthine, shares a similar mechanism of action with caffeine through adenosine receptor antagonism and phosphodiesterase inhibition; however, it has been reported to exhibit lower central nervous system penetration and greater inter-individual variability in neonatal metabolism, which may justify its intermediate clinical response [16,30]. In contrast, doxapram predominantly stimulates peripheral chemoreceptors in the carotid bodies, enhancing respiratory drive by increasing sensitivity to hypoxemia [31]. While this mechanism may produce an immediate respiratory response, its reliance on peripheral chemoreceptor activation may render it less effective in cases of central hypoxic depression. Additionally, previous reports have associated doxapram use with variability in respiratory effort and possible cardiovascular instability in neonates experiencing severe perinatal asphyxia [32] which may account for the broader dispersion of Δ Apgar values. In a recent study with neonatal dogs, there was insufficient evidence that doxapram provided an advantage (or disadvantage) compared to placebo when routinely used in puppies born by elective cesarean section who were not experiencing apnea. Of the 28 puppies that did not achieve an Apgar score of 10, 14 were in the doxapram group and 14 received placebo. There were no significant differences between the Apgar score and non-zero doses of doxapram at each sampling point.

The superiority of caffeine observed in the present study is further supported by cross-species neonatal evidence demonstrating its consistent respiratory and cardiovascular stimulatory effects in mammalian newborns. In foals with experimentally induced respiratory acidosis, caffeine promoted more favorable respiratory and cardiovascular responses than doxapram [33], and subsequent retrospective analysis confirmed that caffeine was more effective than doxapram in correcting hypercapnia in foals with hypoxic–ischemic encephalopathy [34]. In neonatal calves with perinatal asphyxia, caffeine improved arterial blood gases and clinical recovery more effectively than atropine and doxapram [35]. More recently, caffeine administration in neonatal pigs and lambs was reported to enhance cardiopulmonary transition and potentially improve survival rates during the critical postnatal adaptation period [36]. However, it is important to emphasize that when evaluating canine neonates delivered via cesarean section, clinical depression may be greater due to anesthesia and the prominent hypoxemia [1,7,8,9]. Despite this, collectively, these findings reinforce the translational consistency of caffeine as a respiratory stimulant and support the higher clinical responsiveness observed in hypoxic canine neonates in the present study.

The mortality observed in two neonates in the doxapram group and in one in the caffeine group may be associated with prolonged hypoxia in these puppies, which may have developed acute sequelae, such as cell death in various organs, due to reduced tissue oxygenation [12].

The use of the sublingual route for drug administration has been described in human and canine neonates, standing out for its rapid absorption through the mucosa, which is a key pharmacokinetic principle of this route, making it a practical option in emergency management [16,37], especially when intravenous and intraosseous routes are not accessible. The sublingual region has a rich capillary network and veins that drain directly into the internal jugular vein, which in turn connects to the superior vena cava. The most crucial pharmacokinetic characteristic of the sublingual route is that the absorbed drug avoids first-pass hepatic metabolism, resulting in significantly higher bioavailability and a faster time to peak plasma concentration than oral administration, which is crucial in emergency situations such as neonatal resuscitation [38]. The sublingual route is limited to drugs that are potent at low doses because the surface area is small [38]. Administering large volumes of drugs can affect absorption, as it can be influenced by swallowing, leading to slower gastrointestinal absorption and hindering the predictability of pharmacokinetics [37]. The drug doses used in this study were calculated based on doses used for adult dogs [39], and due to the smaller size of the neonate patient, the administered volume was proportionally smaller.

The absence of significant changes in blood gas variables, lactate, glucose, and cardiac troponin I (cTnI) between the different assessment times may be partially attributed to the short monitoring period (10 min) after drug administration. Although the maintenance of arterial pH, oxygen saturation, and carbon dioxide within physiologically acceptable ranges suggests that none of the drugs evaluated induced acute respiratory or metabolic deterioration during initial stabilization, it is possible that cTnI does not reduce its serum level in a short period. In a study that evaluated hypoxic neonatal dogs, troponin I levels were elevated at birth and decreased 1 h later [9]. Future studies should incorporate serial cTnI measurements (e.g., 1–24 h) to better characterize the temporal trajectory of cardiomyocyte injury in relation to clinical recovery.

Some limitations of the present study should be acknowledged. The control group consisted of physiologically normal neonates that did not require resuscitative intervention, rather than a pharmacological placebo group, which may limit the direct comparison of drug-induced effects. Additionally, the relatively small sample size (n = 10 per treatment group) may have reduced the statistical power to detect subtle intergroup differences, particularly in variables with high biological variability. The short evaluation period (10 min post-administration) also restricts the interpretation of delayed pharmacodynamic effects, especially in relation to markers such as cTnI.

The results of this study have potential clinical relevance for neonatal resuscitation in canines. The administration of caffeine may provide veterinarians with a practical, accessible, and evidence-based option to improve immediate neonatal outcomes.

## 5. Conclusions

Caffeine demonstrated the most pronounced improvement in neonatal vitality among hypoxic canine neonates compared with aminophylline and doxapram, suggesting its potential as a more reliable pharmacological adjunct in neonatal resuscitation protocols. Although all evaluated respiratory stimulants promoted some degree of clinical improvement, the greater short-term Δ Apgar response observed with caffeine reinforces its relevance in supporting cardiopulmonary transition during the critical postnatal adaptation period. Studies with larger samples, longer monitoring intervals, and evaluation of medium- and long-term results should be conducted to determine the overall clinical impact of early caffeine administration in neonatal dogs.

## Figures and Tables

**Table 1:** Modified Apgar score for neonatal dogs [1,25].

Parameter/Score	0	1	2
Mucous membrane color	Cyanotic	Pale	Pink
Heart rate	<100 bpm	<200 bpm	200–260 bpm
Respiratory rate	Absent < 6 mpm	Weak and irregular <15 mpm (6–15)	Regular and rhythmic >15 mpm
Muscle tone	Flaccid	Some limb flexions	Flexion
Reflex irritability	Absent	Some movement	Clear crying

bpm = beats per minute; mpm = movements per minute.

**Table 2:** System for evaluating neonatal reflexes in dogs [1].

Indicator/ Score	0	1	2
Sucking	Absent	Poor	Strong
Rooting	Absent	Slow fitting of the snout within the circle	Immediate fitting of the snout within the circle
Righting	Absent (continued in the decubitus position)	Slow body repositioning	Immediate body repositioning

**Table 3:** Mean values of reference parameters for venous blood gas analysis, lactate, oxygen saturation, blood glucose and troponin I of neonatal puppies at birth, from [9].

Parameter	Cesarean Section	
	Non-Hypoxemic Puppies	Hypoxemic Puppies
pH	7.2	7.1
pCO_2_ (mmHg)	49.3	64.4
pO_2_ (mmHg)	13.7	7.0
HCO_3_ (mmol/L)	23.2	25.7
TCO_2_ (mM)	25.1	28.2
Base excess (mmol/L)	−5.1	−4.2
Lactate (mg/dL)	3.9	4.8
SO_2_ (%)	19.6	8.6
Peripheral SO_2_ (%)	97.9	57.6
Glucose (mg/dL)	97.3	122.6
Troponin I (ng/mL)	0.05	0.15

**Table 4:** Maternal and neonatal baseline characteristics of the study population.

Variable	Mean ± SD or n (%)
Number of bitches	14
Maternal age (years)	2.64 ± 0.93
Maternal body weight (kg)	12.64 ± 9.18
Litter size (puppies per bitch)	4.93 ± 3.63 (includes all live-born pups per bitch)
Total neonates’ puppies	69
Total neonates included	45
Neonatal birth weight (g)	267.28 ± 97.34
Neonatal rectal temperature at birth (°C)	33.45 ± 1.65
Sex distribution	♂ 23 (51.1%); ♀ 22 (48.8%)
Early neonatal mortality (first hours)	3 (7.0%)

Data are presented as mean ± standard deviation (SD) or number (percentage). Litter size was calculated based on all live-born pups per bitch, whereas neonatal variables refer exclusively to the 45 puppies enrolled in the study.

**Table 5:** Physiological reference values (mean ± SD) of clinically normal neonatal dogs (control group) five minutes after birth (M1).

Parameter	Mean ± SD
Heart rate (bpm)	196.0 ± 8.98
Respiratory rate (movements/min)	26.10 ± 6.74
Apgar score	10.0 ± 0.00
Reflex score	5.2 ± 0.78
SpO_2_ (%)	98.8 ± 0.42
pH	7.2 ± 0.09
pCO_2_ (mmHg)	48.53 ± 11.07
pO_2_ (mmHg)	15.73 ± 5.93
Base excess (mmol/L)	−4.5 ± 3.17
HCO_3_^−^ (mmol/L)	23.5 ± 2.8
sO_2_ (%)	20.60 ± 10.04
Lactate (mmol/L)	3.41 ± 1.45
Glucose (mg/dL)	93.5 ± 15.52
Troponin I (ng/mL)	0.05 ± 0.01

This group was used as a descriptive physiological reference and was not included in statistical comparisons.

**Table 6:** Clinical parameters (mean ± SD) of neonatal dogs treated with aminophylline, caffeine, or doxapram at birth (M1) and 10 min post-treatment (M10). No significant differences were detected within groups (*p* > 0.05). (Minor discrepancies in sample size among groups resulted from insufficient sample volumes for some biochemical analyses).

Clinical Parameters	Aminophylline Group (n = 12)		Caffeine Group (n = 11)		Doxapram Group (n = 12)		*p* Intragroup
	M1	M10	M1	M10	M1	M10	
Heart rate (bpm)	153.83 ± 22.16	175.00 ± 16.72	147.09 ± 23.82	182.18 ± 12.82	127.38 ± 34.63	156.83 ± 29.48	NS
Respiratory rate (mov/min)	27.00 ± 11.43	23.41 ± 6.38	22.36 ± 11.12	25.63 ± 9.99	19.92 ± 6.75	24.75 ± 5.92	NS
Apgar score	5.50 ± 1.78	8.00 ± 1.12	4.18 ± 1.07	8.45 ± 1.50	4.69 ± 1.43	6.25 ± 1.96	NS
Reflex score	1.25 ± 1.42	3.75 ± 1.71	1.18 ± 1.16	3.36 ± 1.62	1.07 ± 0.95	3.72 ± 1.61	NS
SpO_2_ (%)	98.66 ± 1.15	99.00 ± 0.00	95.54 ± 8.35	98.63 ± 0.92	98.00 ± 3.31	98.91 ± 0.28	NS

Note: NS, not significant; no significant differences were detected between M1 and M10 within each treatment group (*p* > 0.05).

**Table 7:** Δ Apgar score (M10 − M1) in hypoxic neonatal dogs treated with aminophylline, caffeine, or doxapram.

Group	n	Average (Δ) ± DP	Median	Q1–Q2	Min–Max
Aminophylline group	12	2.50 ± 1.31	2.00	1.50–3.50	1–5
Caffeine * group	11	4.27 ± 1.10	4.00	4.00–5.00	2–6
Doxapram group	12	1.64 ± 3.14	2.00	0.00–3.50	5–6

Δ = difference between time points (M10 − M1). Data are presented as mean ± standard deviation (SD), median (Q1–Q3), and minimum–maximum values. Statistical comparison among treatment groups was performed using the Kruskal–Wallis test followed by Dunn’s post hoc test. * Significant difference between caffeine and doxapram groups (*p* < 0.05).

**Table 8:** Blood gas and biochemical parameters (mean ± SD) of neonatal dogs treated with aminophylline, caffeine, or doxapram at birth (M1) and 10 min post-treatment (M10).

Clinical Parameters	Aminophylline Group (n = 12)		Caffeine Group (n = 11)		Doxapram Group (n = 12)		*p* Intragroup
	M1	M10	M1	M10	M1	M10	
pH	7.05 ± 0.10	7.11 ± 0.08	7.02 ± 0.12	7.09 ± 0.09	6.92 ± 0.15	7.08 ± 0.18	NS
pCO_2_ (mmHg)	80.84 ± 13.83	66.80 ± 12.76	88.73 ± 22.02	66.08 ± 15.15	30.58 ± 13.01	66.55 ± 14.17	NS
pO_2_ (mmHg)	29.45 ± 13.35	39.66 ± 14.44	33.0 ± 14.41	55.18 ± 30.41	98.95 ± 18.60	52.0 ± 21.16	NS
Base excess (mmol/L)	−8.51 ± 4.73	−7.95 ± 4.05	−7.75 ± 3.40	−10.12 ± 4.04	−11.8 ± 6.28	−11.13 ± 6.14	NS
HCO_3_^−^ (mmol/L)	22.64 ± 3.55	21.65 ± 3.64	23.20 ± 2.89	19.97 ± 3.28	21.10 ± 4.36	19.20 ± 3.44	NS
sO_2_ (%)	33.90 ± 23.30	50.26 ± 21.50	40.10 ± 23.43	63.56 ± 27.36	31.50 ± 22.54	66.85 ± 22.41	NS
Lactate (mmol/L)	5.19 ± 1.73	5.65 ± 1.63	5.76 ± 1.50	6.73 ± 1.33	7.28 ± 3.18	7.16 ± 1.21	NS
Glucose (mg/dL)	120.58 ± 57.83	120.41 ± 60.31	122.63 ± 48.30	120.81 ± 51.22	135.00 ±51.21	134.66 ± 48.35	NS
Troponin I (ng/mL)	0.07 ± 0.06	0.08 ± 0.05	0.07 ± 0.03	0.08 ± 0.04	0.07 ± 0.04	0.07 ± 0.05	NS

NS: No significant differences were detected between M1 and M10 within each treatment group (*p* > 0.05).

**Table 9:** Comparative summary of pharmacological respiratory stimulation and neonatal recovery outcomes in canine and human neonates.

Study	Species/Condition	Drug(s)/Approach	Outcome Measure	Best Response	Notes/Key Findings
Present study (2025)	Hypoxic canine neonates (cesarean)	Aminophylline vs. Caffeine vs. Doxapram	Δ Apgar (M10 − M1)	Caffeine	Caffeine showed the greatest clinical recovery; two deaths occurred in the doxapram group
Santos et al. (2007) [16]	Canine cesarean neonates	Aminophylline vs. Doxapram (sublingual & SC)	Apgar score	Aminophylline (sublingual)	Sublingual aminophylline led to faster Apgar recovery; doxapram was less consistent
Veronesi et al. (2009) [25]	Canine neonates in different types of delivery	No pharmacological intervention (vitality assessment)	Apgar and reflex recovery over time	--	Routine Apgar evaluation of newborn dogs for assessing viability and determining survival prognoses
Schmidt et al. (2006) [17]	Preterm human neonates	Caffeine vs. Aminophylline	Apnea reduction & survival	Caffeine	Became gold standard in human neonatal therapy
Pereira et al. (2022) [9]	Hypoxic canine neonates	No drug (focus on hypoxia damage)	cTnI elevation, survival	--	Hypoxia associated with myocardial injury; supports need for early intervention
Hyndman et al., 2023 [29]	Puppies delivered elective cesarian	Doxapram	Insufficient evidence	--	insufficient evidence to conclude a difference in the probability of a puppy having an APGAR score
RECOVER Guidelines—Clinical (Boller et al., 2025) [13]	Canine & feline neonates	Standardized neonatal resuscitation algorithm	Viability restoration pathway	PPV + thermal + airway first	Drugs are adjuncts only when ventilation fails
RECOVER Guidelines—Evidence & Gaps (Boller et al., 2025) [14]	Evidence-based gap analysis	Comparative evidence review of stimulant drugs	Evidence strength levels	Caffeine considered promising	Aminophylline moderately supported; doxapram inconclusive; highlights need for comparative trials
Update on Newborn Resuscitation (Boller et al., 2025) [28]	Neonatal canine/feline resuscitation review	Summary of current neonatal stabilization strategies	Survival and revival prospects	Caffeine emphasized	Notes limited species-specific clinical trials on pharmacological stimulants

Source: Present study and previously published data [9,13,14,16,17,25,28,29].

## Data Availability

The data supporting the findings of this study will be made available in the UNESP Institutional Repository upon publication. The raw data supporting the findings of this study are currently held by the authors and will be deposited in the UNESP Institutional Repository after the completion of the dissertation submission process. Until then, the data are available from the corresponding author upon reasonable request.

## Abbreviations

The following abbreviations are used in this manuscript:

AG	Aminophylline Group
CafG	Caffeine Group
DG	Doxapram Group
CG	Control Group
M1	Five minutes after birth (before drug administration)
M10	Ten minutes after drug administration
Δ	Difference between time points (M10 − M1)
bpm	Beats per minute
mov/min	Movements per minute
cTnI	Cardiac troponin I
pO_2_	Partial pressure of oxygen
pCO_2_	Partial pressure of carbon dioxide
BE	Base excess
NaHCO_3_	Sodium bicarbonate
TCO_2_	Total carbon dioxide
SO_2_	Oxygen saturation
SD	Standard deviation
IQR	Interquartile range
°C	Degrees Celsius
mmHg	Millimeters of mercury
ng/mL	Nanograms per milliliter
mg/dL	Milligrams per deciliter
mmol/L	Millimoles per liter
kg	Kilogram
g	Gram
mL	Milliliter
CEUA	Comissão de Ética no Uso de Animais (Animal Use Ethics Committee)
UNESP	Universidade Estadual Paulista “Júlio de Mesquita Filho”
FAPESP	Fundação de Amparo à Pesquisa do Estado de São Paulo
CNPq	Conselho Nacional de Desenvolvimento Científico e Tecnológico
